# Dynamic emission Stokes shift and liquid-like dielectric solvation of band edge carriers in lead-halide perovskites

**DOI:** 10.1038/s41467-019-09057-5

**Published:** 2019-03-12

**Authors:** Yinsheng Guo, Omer Yaffe, Trevor D. Hull, Jonathan S. Owen, David R. Reichman, Louis E. Brus

**Affiliations:** 1 Department of Chemistry, Columbia University, New York, NY 10027 USA; 2 Department of Materials and Interfaces, Weizmann Institute of Science, 76100 Rehovot, Israel

## Abstract

Lead-halide perovskites have emerged as promising materials for photovoltaic and optoelectronic applications. Their significantly anharmonic lattice motion, in contrast to conventional harmonic semiconductors, presents a conceptual challenge in understanding the genesis of their exceptional optoelectronic properties. Here we report a strongly temperature dependent luminescence Stokes shift in the electronic spectra of both hybrid and inorganic lead-bromide perovskite single crystals. This behavior stands in stark contrast to that exhibited by more conventional crystalline semiconductors. We correlate the electronic spectra with the anti-Stokes and Stokes Raman vibrational spectra. Dielectric solvation theories, originally developed for excited molecules dissolved in polar liquids, reproduce our experimental observations. Our approach, which invokes a classical Debye-like relaxation process, captures the dielectric response originating from the incipient anharmonicity of the LO phonon at about 20 meV (160 cm^−1^) in the lead-bromide framework. We reconcile this liquid-like model incorporating thermally-activated dielectric solvation with more standard solid-state theories of the emission Stokes shift in crystalline semiconductors.

## Abstract (web summary)

Lead halide perovskites have unique electronic properties that depend on the crystal’s anharmonicity. Dielectric solvation theories, developed for molecules dissolved in polar liquids, are shown here to reproduce the temperature behavior of carrier solvation in the electronic spectra, implying strongly anharmonic lattice dynamics.

## Introduction

Solution processed lead-halide perovskites (LHPs) are highly promising materials for photovoltaic and optoelectronic applications^1^. Despite their inexpensive and facile synthesis, the lead-halide perovskites exhibit long carrier lifetimes and diffusion lengths, as well as low electron-hole recombination rates^2^. In search of the basic cause of these remarkable properties, several models and perspectives have been discussed, including Rashba splitting^3,4^, ferroelectricity^5–7^, and polaron formation^8–10^. The large polaron model provides a conceptual starting point in understanding the electron-phonon interaction in polar solids^8^. In LHPs, a polaron theory must take into account the strongly anharmonic atomic displacements that are observed at room temperature^11–14^. While marked anharmonicity occurs in some oxide perovskites and other dielectrics^15^, it is unusual in the crystalline semiconductors that are typically employed in optoelectronic device applications. Indeed, semiconductor lattice dynamics and polarons are typically modeled as harmonic with small perturbative anharmonic corrections^16,17^. Recently the significance of the strongly anharmonic lattice dynamics has begun to be recognized and considered in LHPs^18,19^.

In this work we explore the consequence of strong lattice anharmonicity on carrier recombination luminescence. We examine the difference between the absorption and luminescence resonance energies (namely, the emission Stokes shift) as a function of temperature (T). We do so for two typical halide perovskites single crystals: hybrid CH_3_NH_3_PbBr_3_ (MAPbBr_3_) and all-inorganic CsPbBr_3_. The emission Stokes shift stems from the fact that, to a good approximation, absorption instantaneously excites an exciton resonance before any possible excited state lattice relaxation can occur, while emission probes the inter-band electronic transition after lattice relaxation. We observe that in contrast to conventional IV, III-V, and II-VI semiconductors, the emission Stokes shift in lead-halide perovskites significantly increases with T. Using classical dielectric solvation theories, originally developed for describing the Stokes shift of molecules in polar liquids, we reproduce the experimental observation. The efficacy of dielectric solvation theory highlights the role of lattice anharmonicity in carrier-lattice interactions in lead-halide perovskites. Yet we nevertheless show that this result is consistent with the ability of an effective harmonic model to describe the emission Stokes shift.

## Results

### Evolution of lead-halide perovskite optical responses

**Fig. 1:**
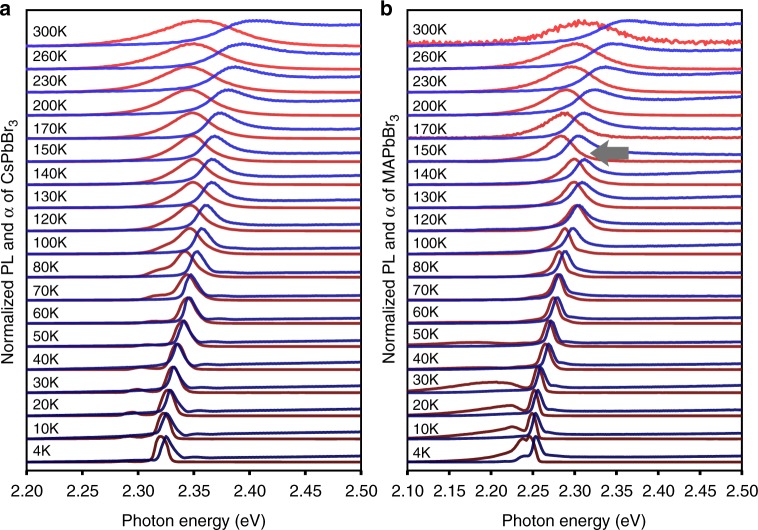
Absorption and photoluminescence from 4 K to 300 K. **a** CsPbBr_3_, **b** MAPbBr_3_. Blue lines show absorption coefficient. Red lines show photoluminescence. Absorption coefficient and photoluminescence spectra at each temperature are scaled and offset vertically for display clarity. Gray arrow indicates the orthorhombic-tetragonal phase transition in MAPbBr_3_

Figure 1 shows the optical absorption and photoluminescence of single lead bromide perovskite crystals. The absorption coefficient *α* is calculated from the observed reflectance spectra using a Kramers–Kronig constrained variational analysis. (Supplementary Notes 1–2 and Supplementary Figures 1–2). Our absorption and emission data agree with that of Tilchin et al.^20^ who made a careful study of MAPbBr_3_ below about 200 K. As previously reported, extrinsic photoluminescence side bands due to shallow traps are observed at low temperatures in MAPbBr_3_^20,21^. The side bands decrease in magnitude at higher T as thermal de-trapping occurs. Furthermore, near 150 K in MAPbBr_3_, an abrupt negative jump in the resonance energy occurs as the orthorhombic to tetragonal phase transition is crossed (gray arrow in Fig. 1b). Overall, the spectral features of MAPbBr_3_ and CsPbBr_3_ and their temperature evolution are remarkably similar.

**Fig. 2:**
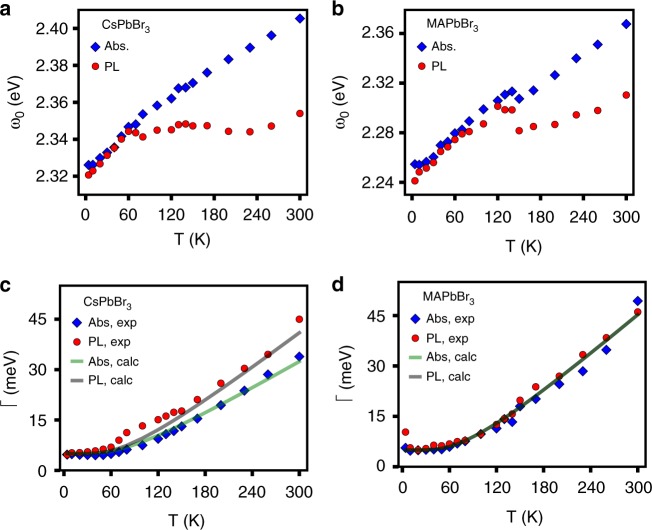
Evolution of spectral features of CsPbBr_3_ and MAPbBr_3_ with temperature. Absorption and emission resonance energies of CsPbBr_3_ (**a**) and MAPbBr_3_ (**b**). Absorption and emission linewidths of CsPbBr_3_ (**c**) and MAPbBr_3_ (**d**). For both materials, blue dots denote the absorption coefficient, *α*, and red dots denote luminescence energies. Green and gray curves show the linewidth evolution predicted by Eq. (1), in which *E*_ph_ = 20 meV, and *γ*_*0*_ = 5 meV. For CsPbBr_3_, *A* = 32 for absorption and *A* = 42 for luminescence. For MAPbBr_3_, *A* = 47 for both absorption and luminescence

Figure 2a, b show the evolution of the peak energy in optical absorption (blue) and photoluminescence (red) for CsPbBr_3_ and MAPbBr_3_, respectively. These results further emphasize the similar behavior of the hybrid and all-inorganic crystals. The resonance energy of the absorption in both materials shifts to the blue as T increases. This blue shift behavior, related to a positive dE_g_/dT, is opposite to that of common semiconductors such as GaAs^22^. However, it is commonly observed in the long-studied lead-based semiconductors^23,24^.

The resonance energy of the photoluminescence (Fig. 2a, b in blue) follows the absorption excitonic resonance up to 50–60 K. At higher temperatures the photoluminescence energy diverges from that of the absorption and becomes almost independent of T, while the absorption excitonic resonance continues to shift blue as it did at lower T. Heuristically, it appears as if the emission no longer follows the band gap physics seen in the instantaneous absorption. We note that the temperature at which the emission behavior breaks from the absorption does not coincide with any known structural phase transition and is well below the tetragonal-orthorhombic phase transition where the organic counter-ions such as in MAPbBr_3_ are known to freeze out^25^.

Figure 2c, d show the evolution of the resonance linewidths in optical absorption (blue) and photoluminescence (red) of CsPbBr_3_ and MAPbBr_3_, respectively. Both absorption and emission resonance linewidths broaden at higher T. Wright et al.^26^ showed that emission broadening in MAPbBr_3_ can be fit by Eq. (1) at higher T. The first term in Eq. (1), *γ*_*0*_, captures temperature-independent broadening factors, such as static inhomogeneities and the intrinsic zero-temperature width, while the second term describes broadening from interaction with a thermal population of modes of energy E_ph_ where *A* is a constant prefactor^26^. The solid curves in Fig. 2c, d show that a phonon energy of about 20 meV reproduces the observed linewidth evolution for both compounds. This observation is in reasonable agreement with that of Wright et al. who observed an activation phonon energy of 15.3 meV (123 cm^−1^). We assign this energy scale to framework LO phonons as did Wright et al.

1 $$\gamma = \gamma _0 + \frac{A}{{\exp \left( {\frac{{E_{ph}}}{{kT}}} \right) - 1}}$$

Both perovskite compounds exhibit similar distributions of framework vibrational modes^11–13,27^. The highest energy optical phonon branch of the lead-bromide lattice occurs at about 20 meV (160 cm^−1^)^28,29^. These modes involve predominantly halide octahedral motion^12,27^. Thus we observe the somewhat expected fact that linewidth broadening shows activated behavior due to carrier coupling to LO phonons.

### Dynamic emission Stokes shifts and dielectric solvation

**Fig. 3:**
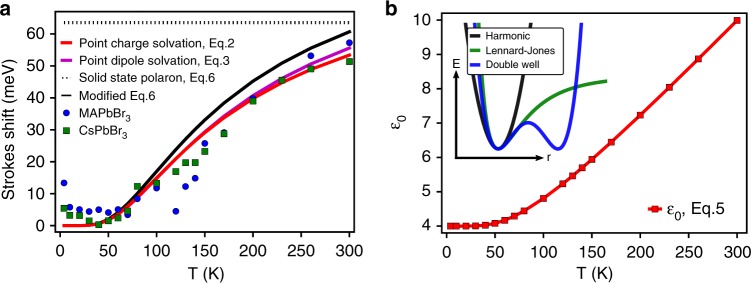
Temperature dependent emission Stokes shift and dielectric response. **a** Emission Stokes shift of CsPbBr_3_ (green squares) and MAPbBr_3_ (blue dots). The two emission Stokes shifts are similar despite their differences in resonance energy and phase sequence. Black dotted line shows the naive prediction from the Fan Eq. (6) with constant *ε*_*0*_. The red curve shows the prediction the point charge dielectric solvation model Eq. (2). The magenta curve shows the prediction the point dipole dielectric solvation model Eq. (3). The black curve shows the revised prediction from the Fan model using the same *ε*_*0*_(T) as in the dielectric solvation models. **b** Temperature dependence dielectric response *ε*_*0*_(T) deduced from spectroscopic data. The insert shows harmonic, Lennard-Jones, and double well schematic potentials. All three curves share the same energy and curvature near the potential minima

Figure 3a shows the evolution of the emission Stokes shift with temperature for CsPbBr_3_ (green dots) and MAPbBr_3_ (blue dots). Below about 50 K the emission Stokes shift is weakly dependent on temperature, and decreases as T increases. This type of Stokes-shifted luminescence is typically produced by potential fluctuations, such as compositional disorder, defect sites, and surface states, which allow relaxation and localization into trap sites before radiative recombination^30^. The influence of such extrinsic fluctuations in the emission Stokes shift decreases at higher temperature as detrapping occurs.

From 60 K to 300 K, the emission Stokes shift strongly increases with T. This finding is both surprising and significant. Generally speaking, an intrinsic emission Stokes shift results from lattice relaxation around an optically excited state. For example, in a Frank–Condon picture, the optically excited state prepared by vertical electronic transition subsequently relaxes to lower vibrational states of the excited potential curve, dissipating the excess energy (the Frank–Condon energy, E_FC_) into phonons. For the simplest model of ground and excited state potential energy curves of displaced harmonic oscillators, the Frank–Condon energy is *E*_*FC*_ = *Sħω*, where *S* is the Huang-Rhys factor and *ħω* is the phonon energy^31^. *S* itself is a geometrical factor with no temperature dependence. In this simple harmonic model the excitonic absorption and emission linewidths will broaden as T increases, but the emission Stokes shift is independent of T. This model, often applied to localized states, clearly does not describe our data.

We now seek to understand the anomalous temperature dependence of the emission Stokes shift. Some temperature dependence should come from the T dependence of the static dielectric constant *ε*_0_. Surprisingly, our observed emission Stokes shift is similar to that measured during solvent relaxation around luminescing molecules in polar liquids^32,33^. When a solute molecule is optically excited in a polar solvent, the solvent molecules will rearrange in response to the altered electrostatic configuration of the solute. At low temperatures the solvent is nearly frozen and a small Stokes shift occurs while at higher temperatures, reorientation is facile. The low frequency (static) dielectric constant of a polar liquid thus shows a large increase from low to high temperature. In typical organic dipolar solvents, a dielectric continuum model captures the non-equilibrium solvation around the excited molecule, as described in the Ooshika-Lippert-Mataga (OLM) relation^34,35^. More general descriptions of this dielectric solvation process exist^36^, which provide closed-form analytical expressions for the emission Stokes shift ${\mathrm{\Delta }}\tilde E(s)$ in the Laplace domain. Here we utilize such models to describe the long time emission Stokes shift via |∆E(∞) − ∆E(0)|. (Details of the implementation are presented in Supplementary Notes 3-5, Supplementary Figures 3–8).

The dielectric relaxation around a single point charge is expressed in Eq. (2), where *s* *=* *iω*, *a* is the radius of a bounding cavity around the solute molecule, and *ε(s)* is the dielectric response of the solvent. In this context, the constant *a* should be no smaller than the excitonic radius. The nature of the luminescing band edge species has been a topic of ongoing discussion^20,37^. We apply this dielectric solvation framework also to dipolar excitonic species. The dielectric solvation of a single point dipole is expressed in Eq. (3), where *p* is the dipole moment of the excitonic species and *v* is the solvation volume, *s* and *ε(s)* are the same as in Eq. (2). The dielectric solvation models for free carriers and excitons capture the experimental observations equally well. (Details are presented in Supplementary Note 4 and Supplementary Figures 6-7).

2 $${\mathrm{\Delta }}\tilde E\left( s \right) = \frac{1}{s}\left( {\frac{{48}}{\pi }} \right)^{\frac{1}{3}}\frac{1}{a}\left( {1 - \frac{1}{{\varepsilon (s)}}} \right)$$

3 $${\mathrm{\Delta }}\tilde E\left( s \right) = \frac{1}{s}\left( {\frac{{8\pi p^2}}{{3v}}} \right)\frac{{\varepsilon \left( s \right) - 1}}{{2\varepsilon \left( s \right) + 1}}$$

4 $$\varepsilon \left( s \right) = \varepsilon _\infty + \frac{{\varepsilon _0 - \varepsilon _\infty }}{{1 + s\tau _D}}$$

A simple description of temperature dependent dielectric response is the Debye dipole relaxation model, expressed in the complex form in Eq. (4). The importance of the difference of the high and low frequency dielectric constants in determining emission Stokes shift is apparent in both the classical Debye model and the solid state Fan model to be discussed below. In Eq. (5) we model the temperature dependence factor in the Debye relaxation, as described in detail in Supplementary Note 4 and Supplementary Figures 6–7. Figure 3b shows the low frequency limit dielectric function ε_0_ obtained by fitting the Eq. (2) and (3) solvation model to the T dependent emission Stokes shift. For both compounds we find that the parameters *A* = 6.5, E_a_ = 20 meV, and *a* = 10 nm reproduce the data in Fig. 3a. The extracted value for E_a_ agrees well with the E_ph_ that was extracted from the linewidth analysis presented in Fig. 2 following Eq.(1).

5 $$\varepsilon _0 - \varepsilon _\infty = \frac{A}{{\exp \left( {\frac{{E_a}}{{kT}}} \right) - 1}}$$

In the dynamics of liquid state processes, it is well-known that the non-equilibrium response of even highly anharmonic systems may be modeled by an effective harmonic model as long as the pertinent fluctuations are Gaussian or if the environment responds in a linear fashion^38–40^. A classical exemplar of this fact is the success of the Marcus theory of electron transfer in polar solvents such as water^41,42^. Indeed, recent simulations demonstrate that even though the perovskite lattice is highly anharmonic, the relevant nuclear fluctuations are statistically nearly Gaussian. Given these facts, we postulate that an effective harmonic phononic model of the Stokes shift may be constructed, using the strongly temperature-dependent dielectric function as input^43^.

From polaron theory, Fan derived Eq. (6) for the emission Stokes shift (from the band edge) of recombining free carriers coupled to harmonic LO phonons^44,45^. Here E_LO_ is the LO phonon energy, and the other symbols have their usual meaning. This model does not consider excitons. This framework works well for GaAs^46^, CdSe^47^, CdTe^48,49^, and other semiconductors at room temperature. In these compounds the emission Stokes shift is just a few meV. Furthermore in such standard cases the static dielectric constant is typically only about 10% larger than the optical dielectric constant^50^. In contrast, the predicted emission Stokes shift (also in Supplementary Note 4 and Supplementary Figures 6–7) for our compounds is large and close to the 60 meV shift we observe; this shift is about three times E_LO_.

6 $${\mathrm{\Delta E}} = \left( {\frac{1}{{\varepsilon _\infty }} - \frac{1}{{\varepsilon _0}}} \right)\left( {\sqrt {\frac{{m_e}}{{m_0}}} + \sqrt {\frac{{m_h}}{{m_0}}} } \right)\sqrt {\left| {E_{Ryd}} \right|E_{LO}}$$

It is useful at this stage to compare the classical dielectric solvation model with the polaron of Eq. (6). In Fig. 3a, the black dashed line shows the naive prediction of Eq. (6) with no temperature dependence. (Parameter values are presented in Supplementary Notes 5–6, Supplementary Figure 8) The magenta curve shows the prediction of a modified Eq. (6), valid at all temperatures, using the low frequency dielectric response in Eq. (5) (Fig. 3b). No adjustment is made to the electron/hole mass or the Rydberg energy, nor is any further fitting of dielectric function performed. Both the T dependence and the absolute magnitude of the emission Stokes shift are captured within this model. From this we learn that the strong T dependence of the emission Stokes shift is rooted in a thermally activated large change of the low frequency dielectric constant.

This dielectric response is far stronger than in the more conventional semiconductors. Thus the coupling between the moving carrier and lattice is large in these lead-halide perovskites. These perovskites show a T dependent dielectric response that behaves roughly as *ε*_*0*_*(T)* ~ *A* *×* *n(T)* where *A* ~ 6 and *n* is the Bose–Einstein population. In conventional semiconductors the dielectric response is *ε*_*0*_*(T)* ~ *A* *×* *T* where *A* ~ 10^−4^ ^50^. This difference is not due to different LO phonon frequencies or populations. For example the E_LO_ of CdTe (21 meV) is very similar to that of CsPbBr_3_ and MAPbBr_3_ (~20 meV). A low E_LO_ and the resulting larger phonon population at higher T does not explain the stronger coupling in lead-halide perovskites.

Polarons form when lattice relaxation around carriers becomes significant^51,52^. Large polarons help to explain the stabilization and protection of carriers in lead halide perovskites^8–10^. Scattering by LO phonons is thought to limit the mobility of polarons^13,53,54^. The dielectric response factor *(1/ε*_*∞*_ *−* *1/ε*_*0*_*)* in Eq. (5) also appears in the Frohlich polaron coupling constant. Our findings suggest that in lead-halide perovskites, the carrier-lattice coupling strength, namely the effective Frohlich coupling constant, is T dependent.

Turning to the structural basis of the dielectric solvation, a large dielectric response emerges from large amplitude, anharmonic nuclear displacements with weak restoring forces. To generate significant dielectric polarization such large displacements are needed. On an anharmonic potential, the dwell time at large nuclear displacement, far away from the low temperature structure, can be dominant. In a heuristic sense this can be visualized by comparing the quadratic, Lennard-Jones, and double-well potential curves shown in the inset of Fig. 3b. These curves have the same harmonic curvature near their potential minima, yet very different displacements and restoring forces at higher potential energies.

Anusca et al. recently described a micellion model in which the rotating MA dipoles organize around a free charge carrier to screen its electric field. In their model, this micellion combines with the standard Frohlich polaron to form a hyperpolaron, which fully accounts for charge carrier screening. While this micellion concept is reminiscent of the solvation-type carrier stabilization we observe, we propose this stabilization has a different structural basis. It is well-known that the MA ions in LHPs are frozen within the orthorhombic phase. The onset of emission Stokes shift take-off occurs at ~60 K, well below the phase transition temperatures. The evolution of emission Stokes shift does not track the structural dynamics of MA rotation, and is almost identical between CsPbBr_3_ and MAPbBr_3_. Nevertheless the MA ion, while neither critical nor necessary, may have a secondary role in solvation. The specific nature of the A site cation does have a clear influence on the abruptness of phase transitions, the microwave and radio frequency range dielectric response, and hot carrier relaxation^10,55–57^.

**Fig. 4:**
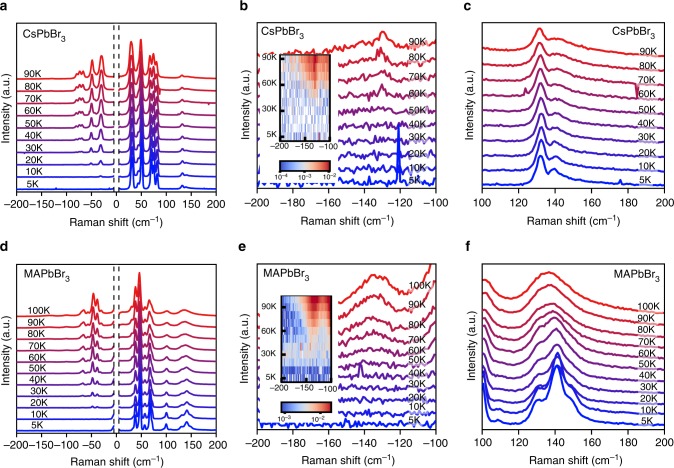
Temperature dependent Raman scattering of CsPbBr_3_ and MAPbBr_3_**. a** CsPbBr_3_. **b** Anti-Stokes and **c** Stokes Raman scattering of the CsPbBr_3_ framework mode just below 150 cm^−1^. **d** MAPbBr_3_. **e** Anti-Stokes and **f** Stokes Raman scattering of the MAPbBr_3_ framework modes just below 150 cm^−1^. Insets in **b** and **e** show the corresponding spectral datasets as color plots

The analysis of both the electronic spectral width and the emission Stokes shift point to significant coupling with the lead-halide framework modes at the top of the phonon dispersion curves (120–160 cm^−1^). Figure 4a, d present the temperature dependent Raman scattering spectra of CsPbBr_3_ and MAPbBr_3_, respectively. Previously we reported their Raman spectra above 80 K^11,27^. The new measurements reported here extend the T range down to 5 K, well below all structural phase transitions. We observe that the Raman modes broaden and shift as they are thermally populated. This thermal population is directly observed in anti-Stokes Raman scattering in Fig. 4b, d. The mode just below 140 cm^−1^ becomes thermally populated near 50–60 K, matching the onset of luminescence Stokes shift rise and linewidth broadening discussed above. Also as discussed above, theoretically the perovskite framework LO mode energies fall just below 150 cm^−1^ at the Brillouin zone center *Γ* point.

### Incipient phonon anharmonicity

The Stokes Raman scattering provides further information about phonon lifetime broadening and the incipient anharmonicity, namely latent nuclear motions that sample an anharmonic potential region as T increases. Figure 4c, f show that the vibrational modes near 140 cm^−1^ are already significantly broadened near 50 K. In contrast, at the same temperature, the modes at lower frequencies remain sharper, as shown in Fig. 4a, d. In a Lorentz oscillator model, the Raman mode becomes broad and asymmetric when the damping term is approximately the same magnitude as the resonance energy. (Details are presented in Supplementary Note 7 and Supplementary Figures 9–10) Such modes are essentially overdamped oscillators; their quantized motion is not firmly established. The broadening of these modes, at the onset temperature of the emission Stokes shift rise, indicates the significance of the incipient lattice anharmonicity. Indeed, local polar lattice fluctuations emerging from large amplitude anharmonic nuclear motions have been reported recently^11,14^. Even at liquid nitrogen temperatures, nuclear motion samples the non-harmonic regions of the potential surface leading to lifetime broadening^11^. Broadening and strong anharmonicity have also been observed in the far IR spectra of MAPbBr_3_ at room temperature^13^. Large amplitude lead-halide octahedral lattice modes responding to photo-generated charge carriers have also been reported on the picosecond to sub-picosecond time scale^9,58,59^.

At low T LHP lattice motions are mostly harmonic and the emission Stokes shift is small. At higher T the anharmonic portion of the potential surface is explored and the emission Stokes shift is large. In more conventional semiconductors, anharmonicity is treated as a perturbative correction to the harmonic approximation, and Raman spectra remain sharp at higher T^17^. In our compounds anharmonicity is well beyond the perturbative regime. The dielectric solvation theory, which we use does not assume a quantized harmonic picture for nuclear motions. Yet note that these equations only consider the “external solvent” polarization in the solid outside the cavity of the luminescing exciton. The question of “inner polarization” inside the exciton certainly merits further investigation.

In summary, we report that luminescing band edge carriers in CsPbBr_3_ and MAPbBr_3_ are stabilized by dielectric response from thermally-activated anharmonic perovskite framework modes. This results in a strongly temperature-dependent emission Stokes shift, whose onset is far below the structural phase transitions. The effective carrier-lattice coupling strength increases at higher temperatures. This behavior is anomalous compared with more conventional crystalline semiconductors. A thermally-activated Debye liquid dielectric solvation model provides insight into this process, and can be used to predict the temperature dependence, as can an effective polaron model for coupling to LO phonons. Vibrational Raman spectra and prior theoretical modeling indicate the dielectric solvation is provided by the halide group modes near 20 meV. A similar dielectric response is observed in both CsPbBr_3_ and MAPbBr_3_. Thus a large dielectric response from strongly anharmonic lattice dynamics is intrinsic to the lead halide perovskite family. Coupling to the orientational dynamics of MA ion is not the dominant effect in carrier recombination luminescence.

## Methods

### Single crystal synthesis

High quality, mm-sized single crystals of MAPbBr_3_ and CsPbBr_3_ were synthesized^11,27^. Briefly, CH_3_NH_2_ (40% w/w Alfa Aesar) and HBr (48% Acros) were mixed to prepare CH_3_NH_3_Br. PbBr_2_ (98% Sigma-Aldrich) and CH_3_NH_3_Br were then dissolved in N,N-dimethylformamide at 1:1 ratio to form 1 M solution. Single crystals of MA were grown by diffusion of isopropyl alcohol vapor into the solution. Single crystals of Cs were grown by diffusion of n-propyl alcohol vapor into a 30 mM solution of 1:1 lead bromide (98%, Sigma-Aldrich) and cesium bromide (99% metal basis, Alfa Aesar) in N,N- dimethylformamide.

### Low temperature optical measurements

Single crystals were mounted onto the cold finger of an optical cryostat. The sample chamber was pumped down to the order of 10^−5^ torr, before cooling down with liquid Helium.

### Optical reflectance spectroscopy

A quartz-tungsten-halogen lamp was used as the white light source. The white light passed through a 100-micron pinhole and was collimated. The white light beam was then sent into an inverted microscope, and imaged onto the back aperture of a ×40 long working distance objective with NA = 0.6. The objective focused the beam onto samples at normal incidence. The reflected light was collected by the same objective and imaged onto the entrance slit of a spectrograph. A 150 grooves/mm grating dispersed the reflected light on a thermoelectrically cooled CCD detector. For each measurement, reflectance of fused silica was collected under the same conditions, which was used as a reference^60^.

### Photoluminescence spectroscopy

Photoluminescence was excited with the 458 nm line of an Ar ion laser. The excitation laser was focused on samples using a ×40 objective with NA = 0.6. Photoluminescence was collected by the same objective, imaged onto the spectrograph entrance slit, and dispersed by a 150 grooves/mm grating on the CCD detector. Typical excitation power was about 0.2 W/cm^2^ at low temperatures (T < 200 K), and about 1 W/cm^2^ at higher temperatures (T > 200 K).

### Low-frequency Raman spectroscopy

The 633 nm line of a Helium-Neon laser was used as the Raman excitation. Spurious light in the laser beam was rejected by a 90/10 volume holographic grating (VHG) beam splitter. The beam was coupled into a home-built inverted microscope, and focused on samples with a ×40 objective with NA = 0.6. The Raman scattered light was collected by the same objective. Rayleigh scattered light was rejected first by the 90/10 VHG beam splitter, and then by two VHG notch filters. Each notch filter has OD > 4 rejection, and a spectral cutoff full width half maximum about 7 cm^−1^ around the 633 nm laser line. After passing through the VHG filters, the Raman signal was spatially filtered, imaged onto the entrance slit of a spectrograph, dispersed by a 1800 grooves/mm grating onto a liquid nitrogen cooled Si CCD detector. Spectral collection covered anti-Stokes and Stokes region from −200 to 200 cm^−1^. The anti-Stokes to Stokes ratio was used to calculate sample spot temperature as expected from the Bose–Einstein distribution. The cryostat temperature control and readout were found to be accurate within ±1 K. Data analysis was done using the Python programming language. Both reflectance and photoluminescence data was fit with the lmfit package^61^.

### Code availability

Custom computer codes used in this work are available from the corresponding author upon request.

## Supplementary information

Supplementary Information

## Data Availability

The data that support the findings of this study are available from the corresponding author upon request.
