# Supplementary material for: Dynamic emission Stokes shift and liquid-like dielectric solvation of band edge carriers in lead-halide perovskites
Source: Nat Commun. 2019 Mar 12;10:1175. doi: 10.1038/s41467-019-09057-5 (PMC6414684; doi:10.1038/s41467-019-09057-5)
Supplement: Supplementary file 1 — Supplementary Information [file 41467_2019_9057_MOESM1_ESM.pdf]

Supplementary Information:

Dynamic Emission Stokes Shift and Liquid-Like Dielectric Solvation of Band Edge Carriers in Lead-Halide Perovskites

Yinsheng Guo,[1] Omer Yaffe,[2] Trevor D. Hull,[1] Jonathan S. Owen,[1] David R. Reichman,[1] and Louis E. Brus[1]*

[1] Department of Chemistry, Columbia University, New York, NY 10027, USA.
[2] Department of Materials and Interfaces, Weizmann Institute of Science, Rehovot, 76100, Israel.
*Corresponding author. Email: leb26@columbia.edu

Supplementary Figures

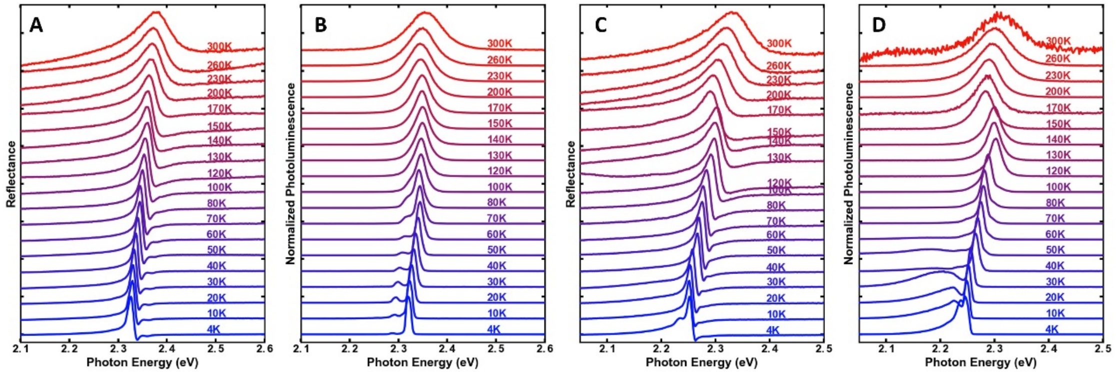

Supplementary Figure 1. Optical reflectance and photoluminescence spectra of $CsPbBr_3$ and $MAPbBr_3$ from 4K to 300K. A. $CsPbBr_3$ optical reflectance, B. $CsPbBr_3$ photoluminescence, C. $MAPbBr_3$ optical reflectance, D. $MAPbBr_3$ photoluminescence. Each photoluminescence spectrum is normalized with respect to its peak intensity. Reflectance and photoluminescence spectra are vertically offset for display clarity.

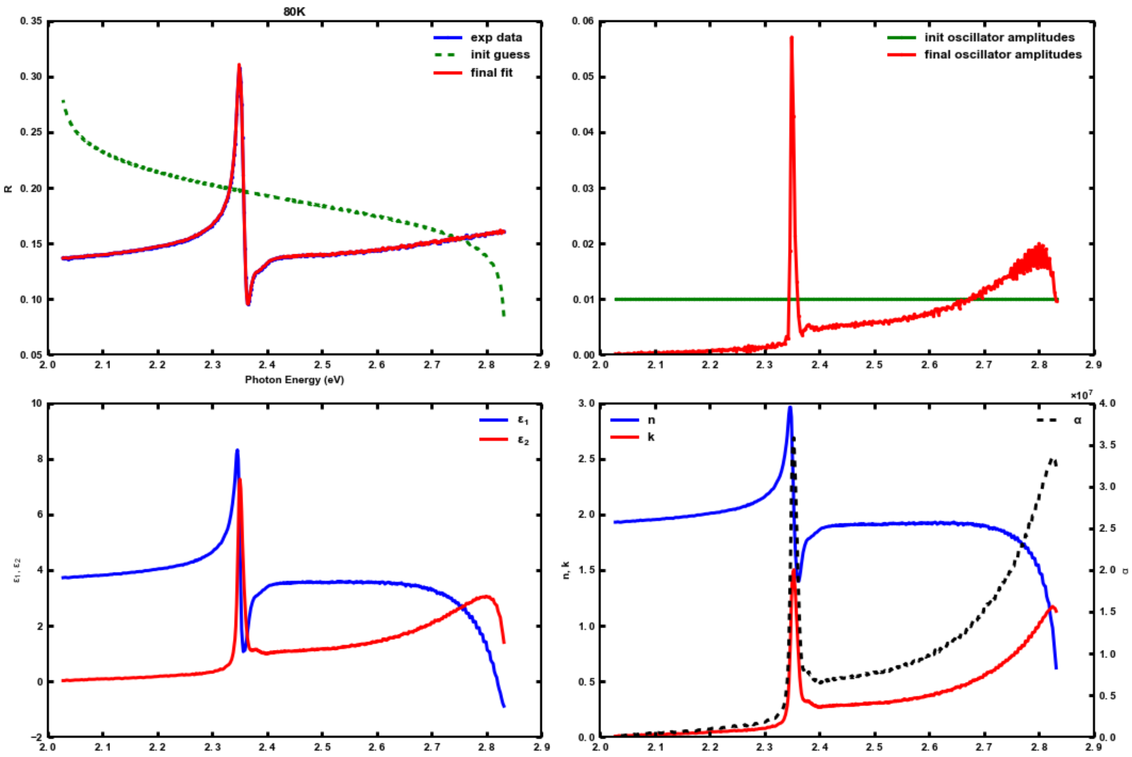

Supplementary Figure 2. Example of Kronig-Kramers constrained variational analysis. Example spectrum was reflectance of $CsPbBr_3$ at 80K.

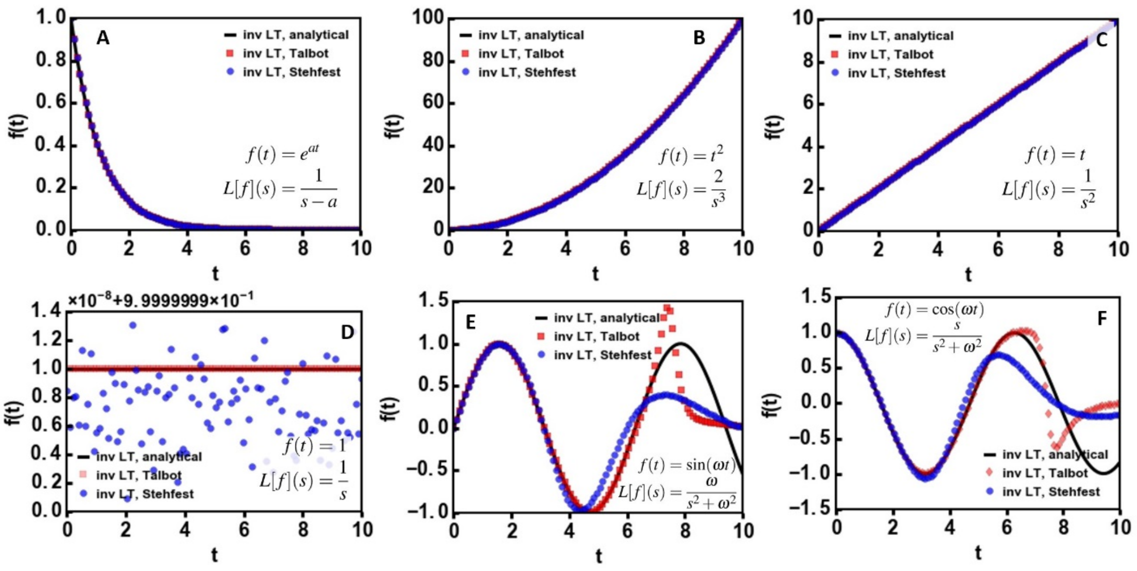

Supplementary Figure 3. Numerical behaviors of inverse Laplace transform algorithms on simple analytical functions.

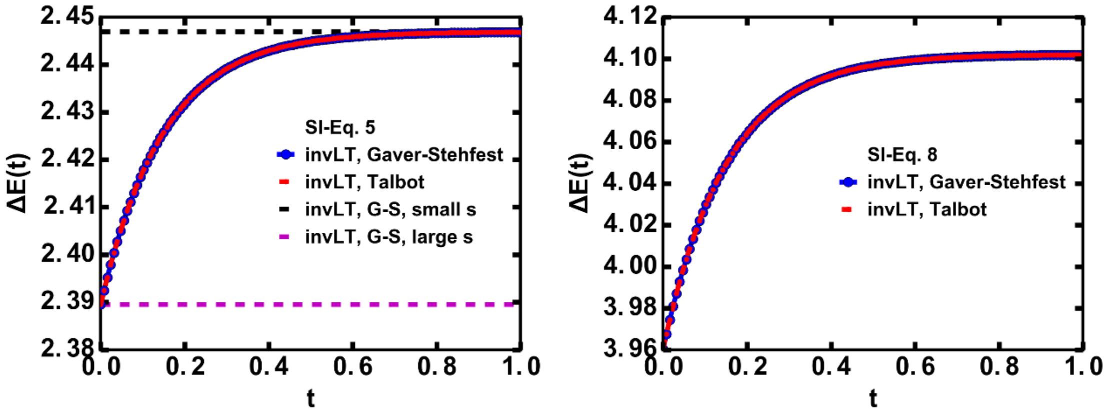

Supplementary Figure 4. Inverse Laplace transform of dielectric solvation dynamics for point charge and point dipole in a dielectric continuum.

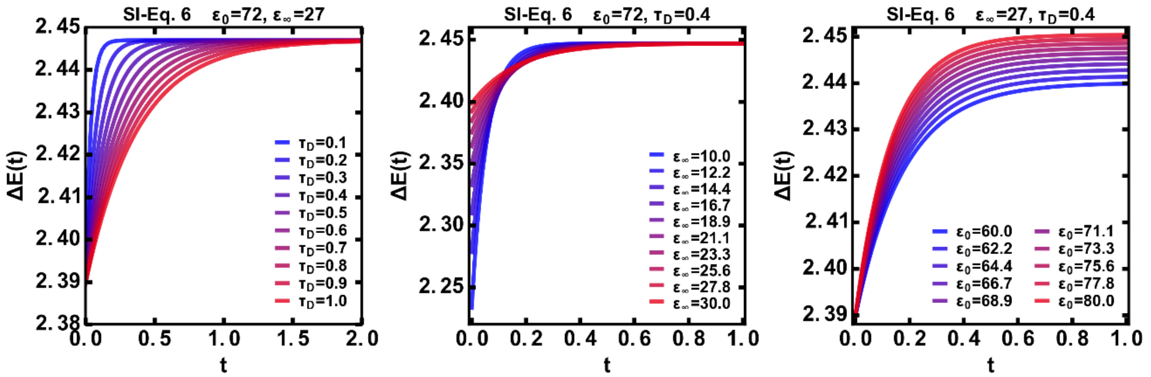

Supplementary Figure 5. Effect of Debye relaxation parameters on the dynamics of dielectric solvation.

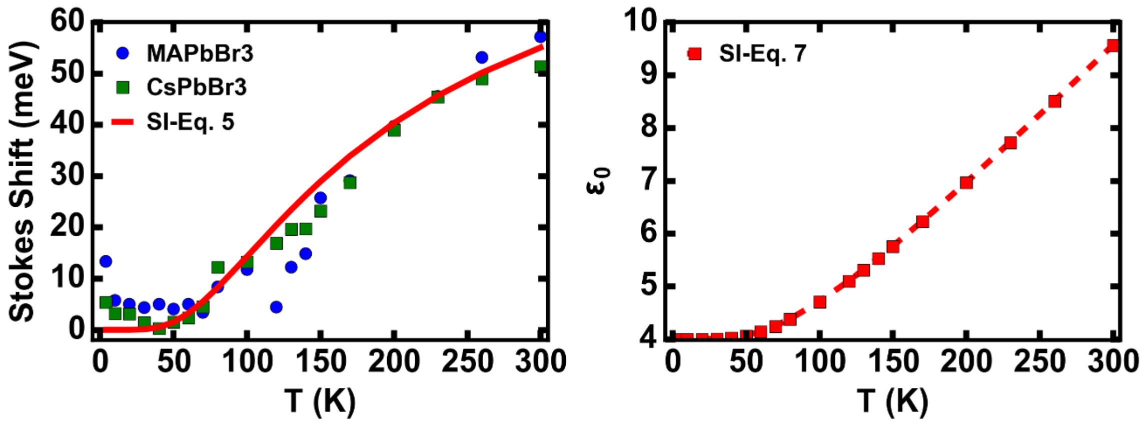

Supplementary Figure 6. Predictions of the point charge solvation model. Left: Point charge solvation model fitting of the T dependent Stokes shift. Blue dots and green squares denote Stokes shift of $MAPbBr_3$ and $CsPbBr_3$ observed in experiments respectively. Red curve shows the Stokes shift predicted via dielectric solvation of point charge. Right: Low frequency limit dielectric function ε_0 revealed by fitting the point charge solvation model to T dependent Stokes shift.

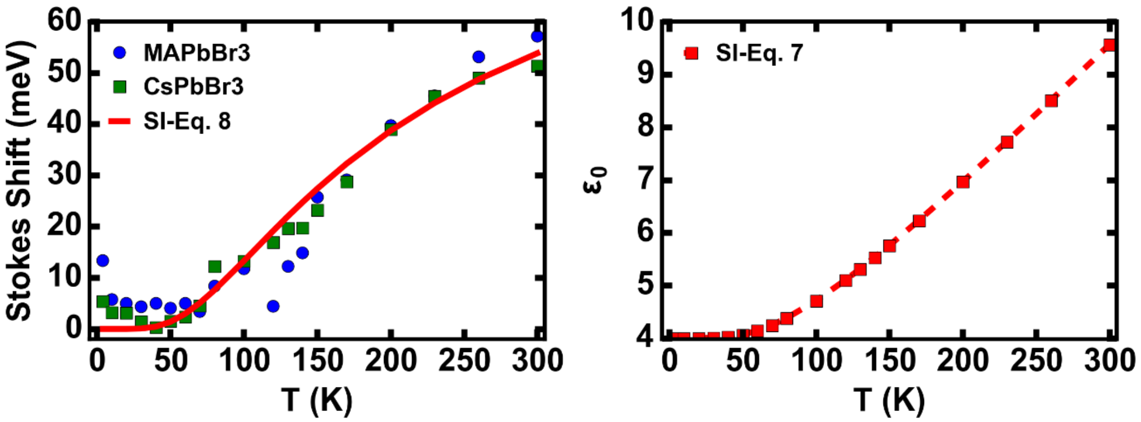

Supplementary Figure 7. Predictions of the point dipole solvation model. Left: Point dipole solvation model fitting of the T dependent Stokes shift. Blue dots and green squares denote Stokes shift of $MAPbBr_3$ and $CsPbBr_3$ observed in experiments respectively, identical to Supplementary Figure 7. Red curve shows the Stokes shift predicted via dielectric solvation of point dipole. The numerical results are very similar to what is shown in Supplementary Figure 7. Right: Low frequency limit dielectric function ε_0 revealed by fitting the point dipole solvation model to T dependent Stokes shift.

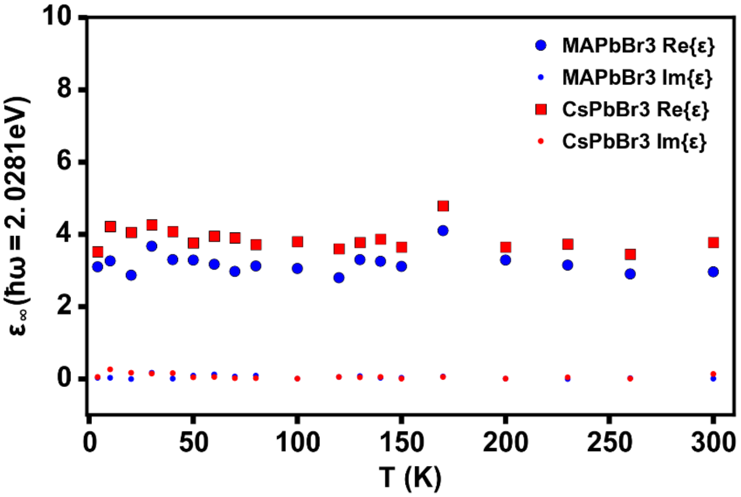

Supplementary Figure 8. Temperature dependence of optical dielectric function below bandgap.

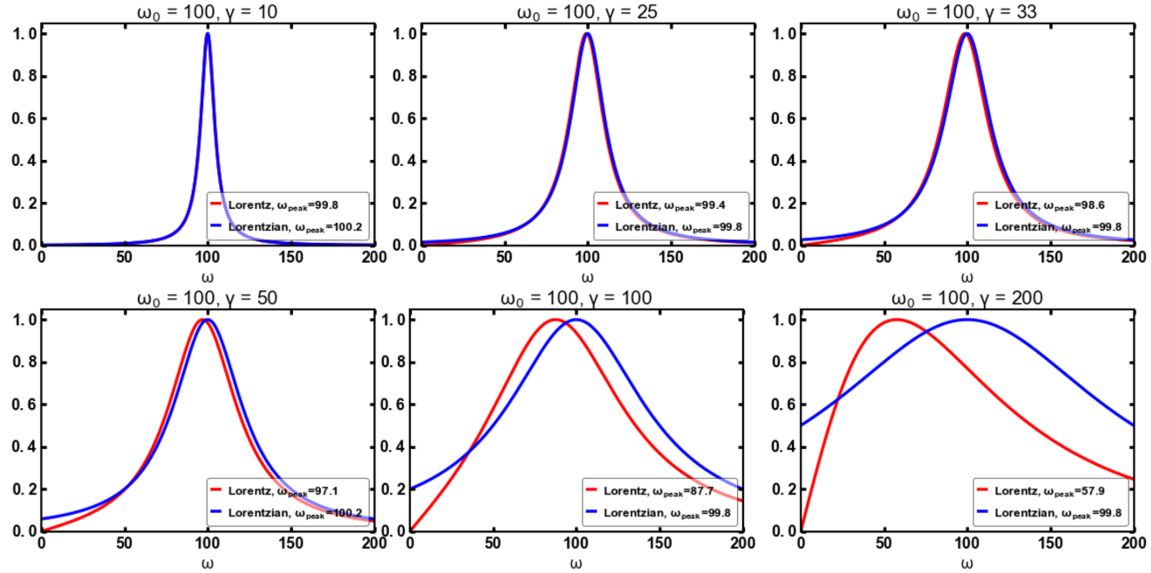

Supplementary Figure 9. Different manifest of damping and anharmonicity in Lorentizan lineshape and Lorentz oscillator models. Blue curves show the Lorentzian lineshapes with a resonance energy of 100 (arbitrary unit) and various damping terms (identical arbitrary unit). Red curves show the behavior of the Lorentz oscillator model with identical parameters. All curves are normalized with respect to the peak intensity.

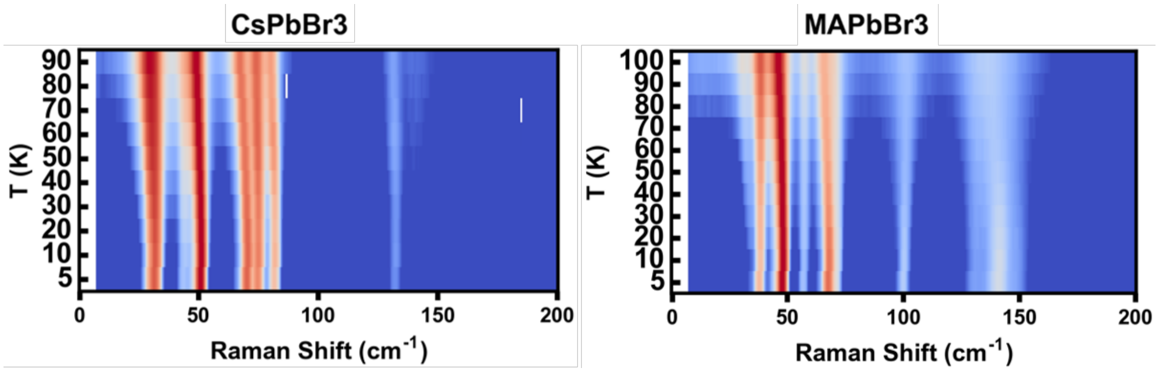

Supplementary Figure 10. Softening and broadening of the low frequency Raman modes of $CsPbBr_3$ and $MAPbBr_3$ at low temperatures. The two color plots display the same data content as shown in Figure 4A and 4D in the main text.

Supplementary Note 1. Analysis of reflectance and photoluminescence spectra.

Supplementary Figure 1 shows optical reflectance and photoluminescence of $CsPbBr_3$ and $MAPbBr_3$ as a function of temperature.

Supplementary Note 2. Kramers-Kronig analysis of reflection.

We calculated the absorption coefficient from the measured reflectance, using Kramers-Kronig constrained variational analysis.[1–3] The key to the calculation of the absorption coefficient was determining the complex dielectric functions using reflectance data, as expressed in Supplementary Equations 1-3. To extract the complex dielectric function, we used a sum of Lorentz oscillators and a constant background, as expressed in Supplementary Equation 4. The use of Lorentz oscillator functional form guarantees that the Kramers-Kronig relationship is valid.

$$R(\omega) = \frac{[n(\omega) - 1]^2 + k(\omega)^2}{[n(\omega) + 1]^2 + k(\omega)^2} \qquad (1)$$

$$\alpha(\omega) = \frac{2\omega}{c} k(\omega) \qquad (2)$$

$$n + ik = \sqrt{\varepsilon} \qquad (3)$$

$$\varepsilon = \varepsilon_\infty + \sum_{k=1}^{N} \frac{\omega_{p,k}^2}{\omega_{0,k}^2 - \omega^2 - i\omega\gamma_k} \qquad (4)$$

Numerically, we used N = 256 Lorentz oscillators, half the number of our available data points in each spectrum. The oscillators were placed at fixed positions, evenly spaced out with 3 meV intervals. The oscillators' width were fixed with γ_k= 5 meV. The oscillator amplitudes and background level were adjustable parameters. This numerical procedure produced a robust representation of the reflectance data.

In Supplementary Figure 2, we show an example of the process, including reflectance spectrum, initial guess values of a regularly spaced, dense forest of peaks, and final spectrum from fitting. Dielectric functions were obtained from the fitting results, according to Supplementary Equation 4. Complex refractive indices and absorption coefficient were obtained, according to Supplementary Equation 3 and Supplementary Equation 2.

We note that, for accurate determination of the optical dielectric function, one has to take into account the contributions from high- and low-lying transitions beyond the measured spectral range. In the fitting the high energy end of the dielectric function showed an unphysical down turn. The complex refractive indices showed the same behavior accordingly. This is due to the absence of higher energy resonances beyond the

spectral range of our data. This apparent lineshape distortion is excluded in subsequent data analysis.

To extract spectral characteristics of the resonance, we fit both the absorption coefficient and photoluminescence resonances with a single Lorentzian function. Spectral background features other than the resonance were modelled with a cubic baseline for absorption and a constant baseline for photoluminescence.

Supplementary Notes 3. Numerical inversion of Laplace transform.

The numerical inversion of a Laplace transform is widely performed.[4] Here we implement the Talbot and the Gaver-Stehfast algorithms. Using simple analytical test function pairs, we test the validity of the inverse transform. As shown in Supplementary Figure 3 A to D, for exponential, quadratic, linear and constant functions, both algorithms return valid results, identical within the numerical precision. As shown in Supplementary Figure 3 E and F, for oscillatory functions both Talbot and Stehfast algorithms fail, and return results that deviate from true values and differ from each other. The occurrence of differences in the inverse transform output can be used to indicate numerical failure and serve as an inherent consistency check.

Next we use the Talbot and the Gaver-Stehfest algorithm to obtain the inverse Laplace transform of the Gaussian field model. The two algorithms are applied to the dielectric solvation processes of a point charge and a point dipole, expressed in Supplementary Equation 5 and 8. The results are shown in Supplementary Figure 4. Both algorithms return the same result in the two scenarios. Moreover, at the limit of very large and very small *s* in the Laplace domain, the Debye relaxation model reduces to the limiting values. The numerical inversion of the Gaussian field model returns the corresponding limiting results consistently.

Having obtained the dielectric solvation dynamics in the time domain numerically, we examine how the dielectric response affects solvation dynamics. Using the Debye relaxation model expressed in the main text with the point charge solvation, we alter the parameters ε_∞, ε_0, and τ one at a time and study the effect of each parameter. The results are shown in Supplementary Figure 5. The parameter τ determines the time constant of the relaxation process. These changes do not affect the extent and strength of screening, only the time needed to reach steady state. The parameter ε_∞ describes the high frequency, or equivalently short time scale, dielectric response. This affects the screening dynamics shortly after excitation, but not the steady state. As ε_∞ increases, $|\Delta E(t = 0)|$ increases, and $|\Delta E(t = \infty)|$ remains constant. The parameter ε_0 describes the low frequency, or equivalently long time scale dielectric response. This affects the screening dynamics long after excitation. As ε_0 increases, $|\Delta E(t = \infty)|$ increases, and $|\Delta E(t = 0)|$ remains constant. We note that this examination treats ε_0, ε_∞, and τ effectively as independent variables. In some physically driven models, ε_0 and/or ε_∞ are often related to τ.[5] The above examination shows that, $\varepsilon_0 - \varepsilon_\infty$ in the numerator is the main factor affecting the steady-state Stokes shift, τ in the denominator has negligible influence on

the steady-state Stokes shift. When $\varepsilon_0 - \varepsilon_\infty$ is temperature dependent, the observed steady-state Stoke shift will thus change as a function of temperature.

Supplementary Notes 4. Gaussian field solvation model fitting of the T dependent Stokes shift and Dielectric function ε0.

Point charge solvation model for fitting the T dependent Stokes shift

As discussed in the text, the Gaussian solvation model provides an analytical solution of the solvation dynamics in the Laplace domain, as shown in Supplementary Equation 5 (Equation (2) in the main text). This is the equation 5.3 in the Song, Chandler, and Marcus paper.[6] Here a is the solvation cell radius, ε(s) is the dielectric function, and s = iω. The dielectric response function of the solvent can be described by a Debye relaxation model, as shown in Supplementary Equation 6, where τ_D is the characteristic time scale of dielectric relaxation. τ_D is sometimes modeled as a function of T and related to $\varepsilon_0 - \varepsilon_\infty$,[5] but it is not always necessary.[7] τ_D as an independent variable is shown to be insignificant in determining steady-state Stokes shift, as discussed in Supplementary Note 3. The temperature dependence of dielectric response can be modelled with Supplementary Equation 7.

$$\Delta\tilde{E}(s) = \frac{1}{s}\left(\frac{48}{\pi}\right)^{\frac{1}{3}}\frac{1}{a}\left(1 - \frac{1}{\varepsilon(s)}\right) \qquad (5)$$

$$\varepsilon(s) = \varepsilon_\infty + \frac{\varepsilon_0 - \varepsilon_\infty}{1 + s\tau_D} \qquad (6)$$

$$\varepsilon_0 - \varepsilon_\infty = \frac{A}{\exp\left(\frac{E_a}{kT}\right) - 1} \qquad (7)$$

Supplementary Figure 6 shows the fitting of the data. In calculation of Supplementary Equation 7 (Equation (5) in the main text) for $\varepsilon_0 - \varepsilon_\infty$, ε_∞ is taken as 4 from experimental data as discussed in Supplementary Note 5, the prefactor A is taken as 6.5, and E_a is taken as 20 meV. The solvation cell radius a is determined to be about 10 nm.

Point dipole solvation model for fitting the T dependent Stokes shift

The nature of the luminescing band edge species at room temperature has been a topic of ongoing discussion. To account for the stabilization of a possible complex formed by bound electron-hole pairs and colliding free carriers, we also model the dielectric solvation of a point dipole. The procedure is similar to that of the point charge. For a point dipole, the analytical solution of solvation dynamics in the Laplace domain is

expressed in Supplementary Equation 8, where p is the dipole moment of excited species, v is the solvation volume, $\varepsilon(s)$ is the dielectric function, and $s = i\omega$. This is the equation 5.7 in the Song, Chandler, and Marcus paper.[6] The description of solvent dielectric response function and its temperature dependence follow from the previous case of point charge solvation, expressed in Supplementary Equation 6 and 7.

$$\Delta\tilde{E}(s) = \frac{1}{s}\left(\frac{8\pi p^2}{3v}\right)\frac{\varepsilon(s) - 1}{2\varepsilon(s) + 1} \qquad (8)$$

Supplementary Figure 7 shows the results calculated from the above model to match experimental data. In calculation of Supplementary Equation 7 for $\varepsilon_0 - \varepsilon_\infty$, ε_∞ is taken as 4 from optical data as discussed in Supplementary Note 5, the prefactor A is taken as 6.5, and E_a is taken as 20 meV, identical to the case of point charge solvation. If one takes the e-h separation as 10 nm, then the required solvation volume is about 2.2×10^3 nm^3. The predicted ε_0 shown here is identical to Supplementary Figure 6.

Supplementary Note 5. Dielectric function in the optical frequency range.

Optical frequency dielectric functions are obtained from the Kramers-Kronig constrained variational analysis as described above. In Supplementary Figure 8, we plot the dielectric function below the main resonance at the low energy end of our spectral range. The dielectric function in this range is largely constant, independent of temperature evolution. Thus we use $\varepsilon_\infty = 4$ for our subsequent analysis of dielectric solvation.

Supplementary Note 6. Prediction of emission Stokes shift based on main text Equation (6)

In the naive prediction of emission Stokes shift based on the solid state Fan model Equation (6) in the main text, we used the following parameters:

m_0: mass of free electron.

E_{Ryd}: Rydberg energy of hydrogen atom in vacuum, 13.6 eV.

m_e: band mass of electron, $0.13m_0$ taken from Miyata et al.[8]

m_h: band mass of hole, $0.19m_0$ taken from Miyata et al.[8]

E_{LO}: the LO phonon energy, 20 meV as obtained from the fitting of emission Stokes shift as well as the fitting of absorption and emission linewidth.

ε_∞: dielectric constant at the high frequency limit, 4.0 as obtained from our optical reflectance measurements.

ε_0: dielectric constant at the low frequency limit, 10.75 as taken from Tilchin et al.[9]

Supplementary Notes 7. Different manifest of damping and anharmonicity in Lorentizan lineshape and Lorentz oscillator models.

The significance of anharmonicity can be better appreciated by recognizing the distinction between a Lorentzian lineshape (Supplementary Equation 9) and a Lorentz oscillator (Supplementary Equation 10). Supplementary Figure 9 compares and contrasts the different behaviors of the Lorentz oscillator and the Lorentzian lineshape with identical resonance energy and damping. As stated in our manuscript, Lorentzian lineshapes are commonly used to capture a resonance, and the approximation is usually assumed good. As the figure shows, when the damping term is below a small fraction of the resonance frequency (weakly anharmonic), the resulting lineshapes do not differ much. When the damping term becomes comparable to the resonance frequency itself (strongly anharmonic), asymmetric change of the lineshape and shift of the maximum occur. Therefore, low frequency resonances are more easily over-damped than the high frequency resonances, given similar damping terms.

$$\mathrm{I}(\omega) = \frac{c}{(\omega - \omega_0)^2 + \left(\frac{1}{2}\gamma\right)^2} \qquad (9)$$

$$\mathrm{I}(\omega) = Im\left\{\frac{c}{\omega_0^2 - \omega^2 + i\omega\gamma}\right\} \qquad (10)$$

With this note, our low frequency Raman results show that the highest frequency (~<150cm^{-1}) lead-halide phonon modes, at low temperature, is significantly broadened and visibly over-damped. The broadening of the ~140cm^{-1} mode groups at the same temperature is stronger than other lower frequency lead-halide modes. These observations clearly evidence the significance of the incipient anharmonicity of these modes at low T, while well within the orthorhombic phase. Additional plots of the low frequency Raman data are shown in Supplementary Figure 10, in which the pronounced broadening of the modes just below 150cm^{-1} is better visualized in contrast with the other lower frequency modes. In addition, the lower frequency modes (below 100cm^{-1}) are also visibly softened within the low T range of 4K to 100K. This is another indication of the significance of the incipient anharmonicity of the lead-halide modes.

Supplementary References

1. Kuzmenko, A. B. Kramers–Kronig constrained variational analysis of optical spectra. *Review of Scientific Instruments* **76,** (2005).
2. Mak, K. F. *et al.* Tightly bound trions in monolayer MoS2. *Nat Mater* **12,** 207–211 (2013).
3. Li, Y. *et al.* Measurement of the optical dielectric function of monolayer transition-metal dichalcogenides: MoS2, MoSe2, WS2, and WSe2. *Phys. Rev. B* **90,** 205422 (2014).
4. Abate, J. & Whitt, W. A Unified Framework for Numerically Inverting Laplace Transforms. *INFORMS Journal on Computing* **18,** 408–421 (2006).
5. Homes, C. C., Vogt, T., Shapiro, S. M., Wakimoto, S. & Ramirez, A. P. Optical Response of High-Dielectric-Constant Perovskite-Related Oxide. *Science* **293,** 673–676 (2001).
6. Song, X., Chandler, D. & Marcus, R. A. Gaussian Field Model of Dielectric Solvation Dynamics. *J. Phys. Chem.* **100,** 11954–11959 (1996).
7. Frohlich, H. *Theory of dielectrics; dielectric constant and dielectric loss.* (Clarendon Press, 1949).
8. Miyata, K. *et al.* Large polarons in lead halide perovskites. *Science Advances* **3,** (2017).
9. Tilchin, J. *et al.* Hydrogen-like Wannier-Mott Excitons in Single Crystal of Methylammonium Lead Bromide Perovskite. *ACS Nano* **10,** 6363–6371 (2016).
